# Transcriptome Profiling of Two Ornamental and Medicinal *Papaver* Herbs

**DOI:** 10.3390/ijms19103192

**Published:** 2018-10-16

**Authors:** Jaehyeon Oh, Younhee Shin, In Jin Ha, Min Young Lee, Seok-Geun Lee, Byeong-Chul Kang, Dongsoo Kyeong, Dowan Kim

**Affiliations:** 1 Genomics Division, National Institute of Agricultural Science, RDA, 370, Nongsaengmyeong-ro, Wansan-gu, Jeonju-si 54874, Jeollabuk-do, Korea; jhoh8288@korea.kr; 2 Data Science Center, Insilicogen Inc., Yongin-si 16954, Gyeonggi-do, Korea; yhshin@insilicogen.com (Y.S.); bckang@insilicogen.com (B.-C.K.); dskyeong@insilicogen.com (D.K.); 3 Department of Biological Sciences, Sungkyunkwan University, Suwon 16419, Korea; 4 Korean Medicine Clinical Trial Center (K-CTC), Kyung Hee University Korean Medicine Hospital, Seoul 02447, Korea; ijha0@naver.com (I.J.H.); papermint221@gmail.com (M.Y.L.); seokgeun@khu.ac.kr (S.-G.L.); 5 KHU-KIST Department of Converging Science & Technology, Kyung Hee University, Seoul 02447, Korea

**Keywords:** poppy, *Papaver rhoeas*, *Papaver nudicaule*, transcriptome, alkaloid biosynthesis, target metabolome

## Abstract

The *Papaver* spp. (*Papaver rhoeas* (Corn poppy) and *Papaver nudicaule* (Iceland poppy)) genera are ornamental and medicinal plants that are used for the isolation of alkaloid drugs. In this study, we generated 700 Mb of transcriptome sequences with the PacBio platform. They were assembled into 120,926 contigs, and 1185 (82.2%) of the benchmarking universal single-copy orthologs (BUSCO) core genes were completely present in our assembled transcriptome. Furthermore, using 128 Gb of Illumina sequences, the transcript expression was assessed at three stages of *Papaver* plant development (30, 60, and 90 days), from which we identified 137 differentially expressed transcripts. Furthermore, three co-occurrence heat maps are generated from 51 different plant genomes along with the *Papaver* transcriptome, i.e., secondary metabolite biosynthesis, isoquinoline alkaloid biosynthesis (BIA) pathway, and cytochrome. Sixty-nine transcripts in the BIA pathway along with 22 different alkaloids (quantified with LC-QTOF-MS/MS) were mapped into the BIA KEGG map (map00950). Finally, we identified 39 full-length cytochrome transcripts and compared them with other genomes. Collectively, this transcriptome data, along with the expression and quantitative metabolite profiles, provides an initial recording of secondary metabolites and their expression related to *Papaver* plant development. Moreover, these profiles could help to further detail the functional characterization of the various secondary metabolite biosynthesis and *Papaver* plant development associated problems.

## 1. Introduction

Plants in the *Papaver* genus from *Papaveraceae* family (commonly known as poppy) have been used in traditional medicinal practices for a long time. They are also famously known for their alkaloid extract, which is among the constituents of addictive drugs [1]. Historical medical evidence shows that *Papaver* spp. have been used since Greek and Roman times (293–185 BC) [1], and *Papaver* spp. cultivation is mentioned in Sumerian cuneiform script dating from ~3000 BC [2,3]. Moreover, in Korean history, *Papaver* spp. was used as a red drug, and it played a crucial role during the Japanese colonization of Korea [4]. Furthermore, the transformation of *Papaver* from traditional practices to current systems has been described in more detail by Barceloux, D.G and Bernath, J. [2,3]. Furthermore, the related medical/general usage regulations and drug abuse are being monitored by a separate institution (United Nations office on drugs and crime) to prevent overuse [5]. However, there have been minimal research finding on various aspects of the *Papaver* genus (61 species and nine subspecies [6]). *Papaver somniferum* and *Papaver setigerum* are controlled species in Korea [7], and other species are used for ornamental and medicinal purposes. The *Papaver rhoeas* (Corn poppy) and *Papaver nudicaule* (Iceland poppy) species are widely used for ornamentation due to their multi-colored flowers and different shapes. Furthermore, the flower of *Papaver rhoeas* is the symbol for Remembrance Day (or Poppy Day) [8], and it is distributed widely around the world, e.g., Europe, Asia, America, and Africa [8,9].

*Papaver* spp. has various functional benefits that are widely used to treat various issues, such as dental problems [10], neuronal disorders [11], cough [12], pain relief [13], microbial infections [14], and cancer [15]. Some species’ seeds, pedicles, and petals are used in salads or sorbets [16]. Furthermore, *Papaver* is not only beneficial for humans, but also helpful to pollinators. Notably, its pollen and nectar are consumed by pollinators and provide essential sugars and protein [17]. Therefore, *Papaver* species are used to retain the insect biodiversity in isolate settings [17]. Interestingly, the pollen collected by honey bees is used in folk medicine to treat various symptoms [18]. Other than these uses, *Papaver* plants are widely used as a raw material in industries to isolate alkaloid drug components [19], and so far, more than 170 alkaloids have been isolated from these species [20]. In *Papaver rhoeas*, the primary drug components are rhoeadine, iso-rhoeadine, protopine, and latericine [7], and in *Papaver nudicaule*, the primary drug components are nudicaulins [21]. Further, the industrial waste from *Papaver* plants contains highly volatile chemicals that are more feasible for the production of biofuels [22]. Furthermore, *Papaver* contributes its self-incompatibility molecular mechanisms to other plants [23,24,25], and it is used as a model plant for studying non-target site resistance to herbicides, which is one of the original problems in chemical weed control systems [26].

In the pharmaceutical industry, multiple drugs are synthesized from plants by engineered microbes. Similarly, specialized opiate biosynthesis systems have been constructed and optimized in *Escherichia coli* [27] and yeast [28] from *Papaver*. However, the *Papaver*-specific metabolite papaverine and its corresponding biosynthetic cascade and other secondary metabolic cascade enzymes mostly remain to be characterized due to the scarcity of genetic material [29]. Another view, *Papaver*, has six developmental stages i.e., (1) dormant, (2) germination, (3) leaf rosette, (4) branching and elongation of internodes, (5) blossom to seed formation, and (6) seed ripening [3]. Among those leaf rosette stage holds long period of time in *Papaver* developmental cycle. Mainly, the production of alkaloids is highly connected with cell differentiation process and the alkaloid accumulations were correlated with *Papaver* developmental life cycle [3]. Furthermore, the optimum alkaloids accumulation points also identified at two developmental points, i.e., before bud formation and at start of the flowering [3]. To understand this in more detail, the transcriptome data-set is not available. Although, a few studies used high-throughput sequencing, such as 454 [8,30,31] and Illumina [29], but this was not sufficient to characterize the gene content in *Papaver* species.

With this knowledge, this study was further modeled to capture the transcripts expression patterns and alkaloid accumulation at three different time points in *Papaver* developmental life cycle, from stage three to five. The three-time points are leaf rosette (30 days), elongation and branching (60 days), and blossom and seed formations (90 days). Although the other organ of this plant hold higher medicinal value, still we have chosen leaves to observe the transcripts expression and differential expression profiles at different developmental stages. Moreover, we have reported the first study on the generation of the transcriptome from long-read sequencing technology and an assessment of differential transcript expressions during the *Papaver* plant developmental stages. These transcripts were compared with 51 closely- and distantly-related plants from monocots and dicots to obtain an overview of the secondary metabolites, with reference to the KEGG database. These transcripts and metabolites are responsible for isoquinoline alkaloid biosynthesis (BIA), which is marked in the BIA pathway (KEGG map id: map0500), along with the expression, and the full-length cytochromes were predicted and observed with differential expressions. In our study, we have profiled 860 secondary metabolite biosynthesis transcripts, 39 cytochromes, and 69 BIA synthesis transcripts, with their relative expressions from three different plant developmental stages, i.e., from leaf rosette stage to blossom and seed formation stage.

## 2. Results

### 2.1. De Novo Transcript Assembly and Annotations

**Figure 1:**
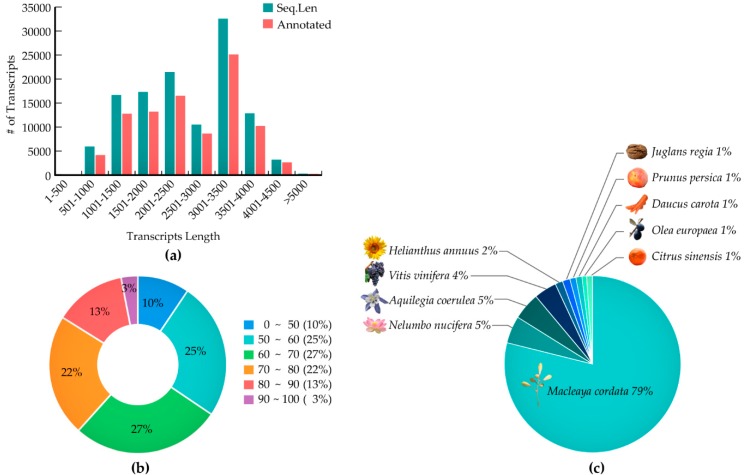
Illustration of the transcriptome assembly and functional annotation summary; (**a**) length distribution of assembled transcripts with and without annotations; (**b**) distribution of BLAST similarities; and, (**c**) species distribution from the BLAST outputs (all the images used in this figure are released free of copyright under the Creative Commons CCO license).

To obtain the full-length transcripts of the *Papaver* species *Papaver rhoeas* and *Papaver nudicaule*, we have sequenced 700 Mb from the PacBio sequencing. After filtering out the low-quality reads and adapter contamination according to the technologies that were used (Table 1, section A), a total of 302 Mb high-quality long read sequences were assembled into 120,926 contigs with 3117 bases of N50 (called the representative transcriptome) (Table 1, section A). Most of the sequence lengths were between 3000 and 3500 bp (Figure 1A). This representative transcriptome contains 1185 (82.3%) benchmarking universal single-copy orthologs (BUSCO) genes present in the reference transcriptome, and only 180 (12.5%) genes were missing from the representative transcriptome (Table 1, section D). 

The absence of these genes can be explained by various reasons, for example, the sequence coverage was not enough and those genes could only be expressed in a very low quantity [32]. Furthermore, the transdecoder has translated 85.2% of the transcripts into proteins, and, among those, 68.9% were complete (Table 1, section C). The representative transcriptome was annotated with the Uniprot plant reference database, and 77.5% of the transcripts has obtained an annotation. Approximately 39% of the sequences had a sequence identity of 70 to 100% to the Uniprot plant reference database (Figure 1B), meaning that most of the annotated transcripts could share reliable functional annotations from the reference sequences. Seventy-nine percent of the annotations was obtained from *Macleaya cordata* (Figure 1C), which is the only species in the *Papaveraceae* family for which a draft genome sequence is available. Finally, in an effort to more precisely characterize the annotations, we found that 57.4% of the representative transcripts were assigned to a gene ontology term (GO) and only 11% of the representative transcripts were assigned to a KEGG enzyme (Table 1, section B).

### 2.2. Differentially-Expressed Transcripts

**Figure 2:**
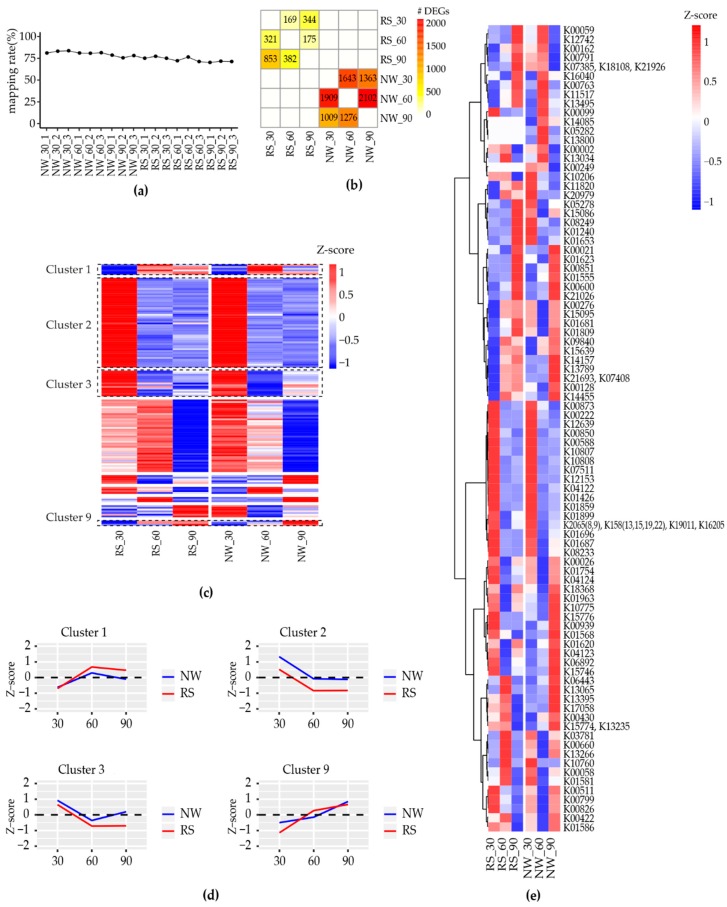
Overview of the differential expression patterns obtained from the given conditions: (**a**) individual sequence library reads mapped to the representative transcriptome; (**b**) quantitative summary of the differentially expressed transcripts concerning plant developmental stages; (**c**) a total of 137 differential expressed transcripts from all given pairs of conditions and their selected *K*-means hierarchical clusters, upon the desired expression patterns; (**d**) selected clusters from 137 common differentially expressed transcripts; and, (**e**) secondary metabolite transcripts which are involved in differential expression. Here, the transcripts were grouped into KEGG ortholog IDs, and the average expressions are shown while multiple transcripts belong to same ortholog ID. NW: *Papaver nudicaule*, RS: *Papaver rhoeas*.

To assess the transcripts expression patterns concerning the plant developmental stages, three-time points, i.e., 30, 60, and 90 days leaves were sequenced. Three biological replicates were sequenced for each sample using the Illumina HiSeq paired-end assessment. Altogether, 128.8 GB of RNA-Seq data were obtained from 18 given samples. After filtering out low-quality reads and adapter contaminations, 76.8% (an individual summary is given in Figure 2A) of the Illumina reads were mapped to the representative transcriptome, and differential expression was calculated while using transcripts per million (TPM). A total of 5421 transcripts showed differential expression patterns in at least one pair of given conditions (an individual summary is given in Figure 2B; additionally, see Supplementary File S1), and, among those, 137 transcripts were noted to have differential expression in both species (Figure 2C). Furthermore, these transcripts were grouped into nine clusters, and four clusters were selected according to the desired expression patterns (Figure 2D). 

In cluster one, the expression of two transcription-responsible transcripts (PSIISO0021193: AP2-ERF domain and PPNISO0037702: NAC domain) depends highly on the growth stage. In cluster two, most of the differentially-expressed transcripts were involved in the transcription mechanism, and notably, in one cytochrome, they were responsible for brassinosteroid biosynthesis (PPRISO0050638). In cluster three, most grouped transcripts show the transcription factor functions. The transcripts that were responsible for the biosynthesis of sesquiterpenoid and triterpenoid (PSIISO0046781) were also grouped in the third cluster. Finally, cluster nine contains the transcript responsible for peptidase-C1A and glycosyl-transferase. Furthermore, another 137 (from the total 5421 DGEs) transcripts showing differential expression are involved in the biosynthesis of secondary metabolites, and most of these transcripts are involved in the biosynthesis of antibiotics and phenylpropanoids (Figure 2E; additionally, see Supplementary File S1).

### 2.3. Secondary Metabolite Biosynthesis Transcript Profiles

**Figure 3:**
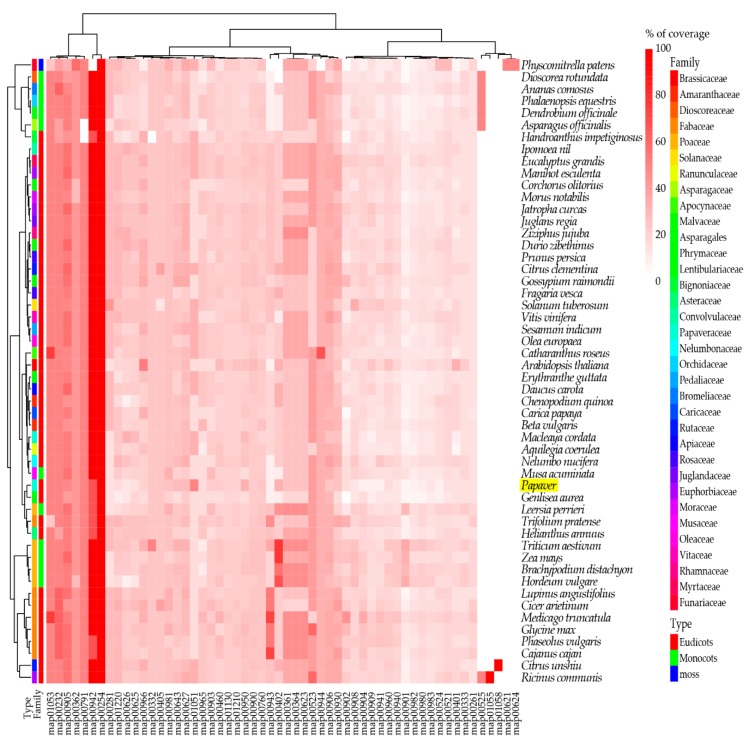
Overview of secondary metabolite biosynthesis pathways and its coverage. Here the transcripts were linked to the KEGG orthologs (KO), by assessing the sequence similarity between KOs and *Papaver* transcripts, which was obtained from the ortholog analysis. The coverage of each pathway was calculated and plotted into the percentage scale (0 to 100%). Here, the coverage was calculated by the formula (Coverage per each KEGG map = (Number of KOs present in transcriptome/Total number of KOs) × 100).

To understand the presence of secondary metabolite biosynthesis transcripts/genes in *Papaver* species and in 51 other plant genomes (34 families; 12 monocots, 20 eudicots, and one moss), a comparative heat map was derived (Figure 3). 

**Figure 4:**
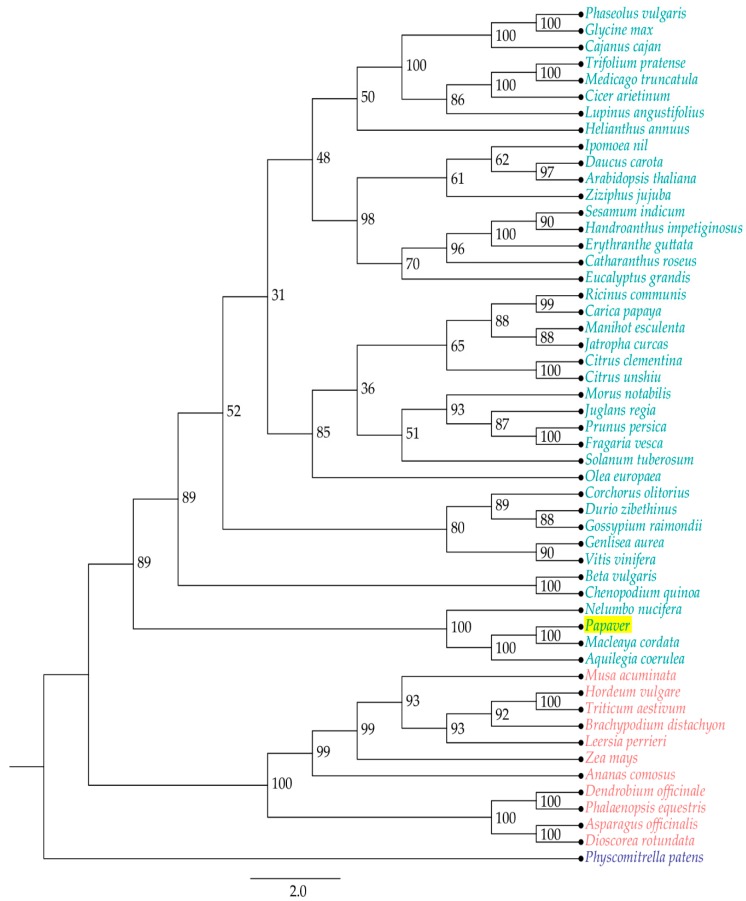
The phylogenetic tree reconstructed to understand evolutionary relationship between *Papaver* and other selected 51 genomes (see Figure 3 for more details) with respect to secondary metabolite transcripts. This tree constructed with 161 single-copy secondary metabolite biosynthesis responsible transcripts, which includes their isoforms. This tree was re-constructed from 1000 bootstrap trees.

Genomes were selected based upon three characteristics—flower colors, edible fruits, and general food/oil crops—along with the model plants. Among these genomes, 56 KEGG secondary metabolite biosynthesis pathway heat maps were derived from the ortholog analysis. The heat map includes 860 transcripts from the *Papaver* representative transcriptome, which was grouped into 580 clusters and 161 single-copy genes. To understand the evolutionary relationship among the given genomes regarding the secondary metabolite biosynthesis, a phylogenetic tree was reconstructed from a concatenation of 161 single-copy gene/transcripts that were extracted from the above clusters (Figure 4).

In the tree, the *Papaver* genus was grouped with three genomes: *Macleaya cordata*, *Aquilegia coerulea*, and *Nelumbo nucifera*. Among these genomes, *Macleaya cordata* [33] and *Nelumbo nucifera* [34] are rich in alkaloid contents and, recently, the *Aquilegia coerulea* genome was studied due to its adaptive radiation within its subspecies [35]. Furthermore, 137 secondary metabolite biosynthesis transcripts that are differentially expressed during *Papaver* plant developmental stages (Figure 2E) showed the potential to be responsible for the concentration variation of secondary metabolites during the *Papaver* plant developmental stages. Through this heat map, we have observed that the two pathways in *Papaver* (biosynthesis of ansamycins (map01051), isoquinoline alkaloid biosynthesis (map00950)) have different gene coverage compared to the others. Other than the *Papaver* transcriptome, this heat map facilitated more observations that were more specific to the genomes, i.e., biosynthesis of vancomycin group antibiotics (map01055) was more specific to *Ricinus communis* and acridone alkaloid biosynthesis (map01058) was more specific to *Citrus unshiu*. Furthermore, some maps are more specific to the higher clades of flowering plants, i.e., Acarbose and validamycin biosynthesis (map00525) was more specific to some monocots (five genomes) than eudicots, whereas only one genome in the eudicots (*Ricinus communis*), as well as maps dioxin degradation (map00621) and polycyclic aromatic hydrocarbon degradation (map00624), were more specific to moss (*Physcometrella patens*).

### 2.4. Biosynthesis of Isoquinoline Alkaloid (BIA) 

**Figure 5:**
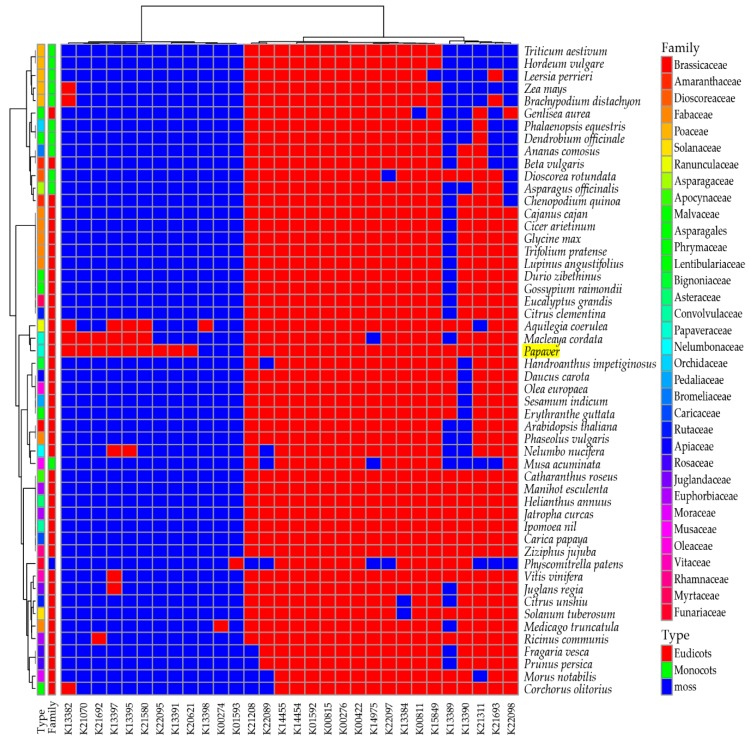
Overview of the isoquinoline alkaloid biosynthesis pathway (map00950). Here the transcripts were linked to the KEGG orthologs (KO), by assessing the sequence similarity between KOs and *Papaver* transcripts, which was obtained from the ortholog analysis. The expression of the heat map is shown in red (transcripts present) and blue (transcripts absent), this information is obtained from the ortholog clusters.

**Figure 6:**
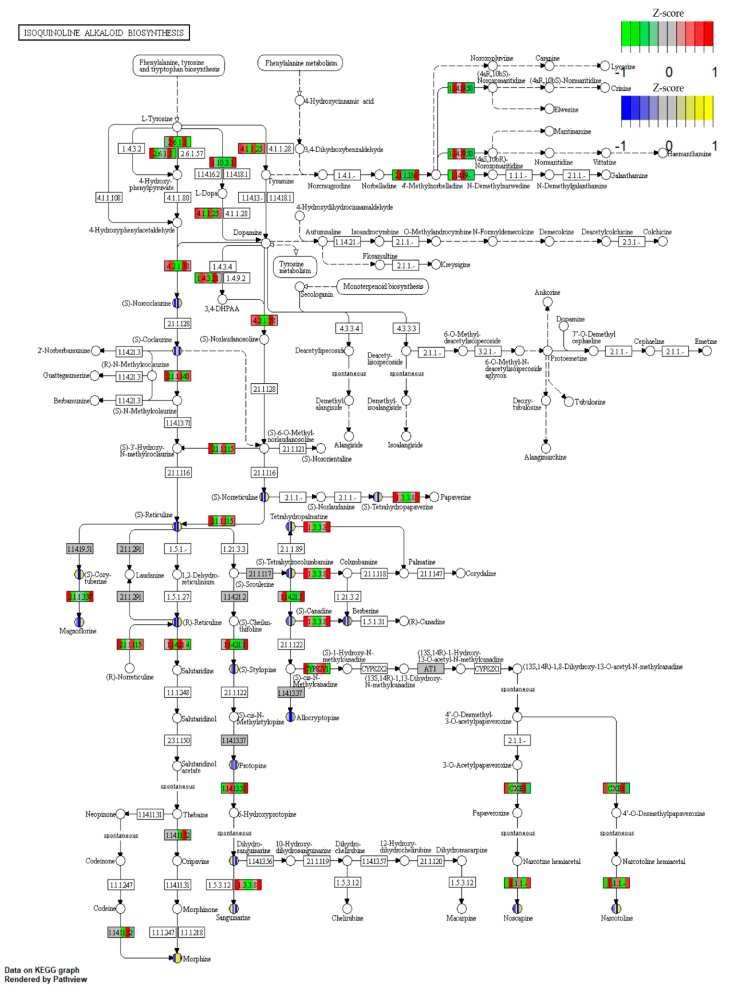
Overview of the isoquinoline alkaloid biosynthesis pathway (KEGG Pathway ID: map00950). The map is marked with the available transcripts and metabolites along with their differential expression patterns using Pathview. The color codes for the transcript differential expression values were in green and red, and for the metabolite, differential quantity values were in blue and yellow color. The sample order is RS (30,60,90), and NW (30,60,90).

**Figure 7:**
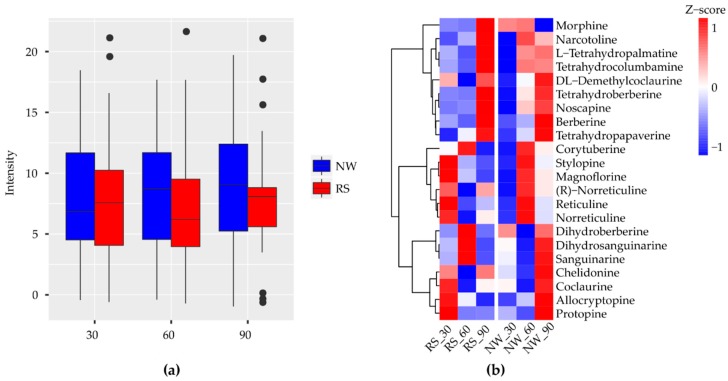
The quantification of alkaloids observed from the target metabolite analysis; (**a**) the total alkaloid content assessed from the *Papaver* plant developmental stages; (**b**) The detail expression of individual metabolites with respect to *Papaver* spp. developmental stages, which used to derive the total metabolite quantifications and these metabolites were mapped to the BIA KEGG reference map (Figure 6).

**Figure 8:**
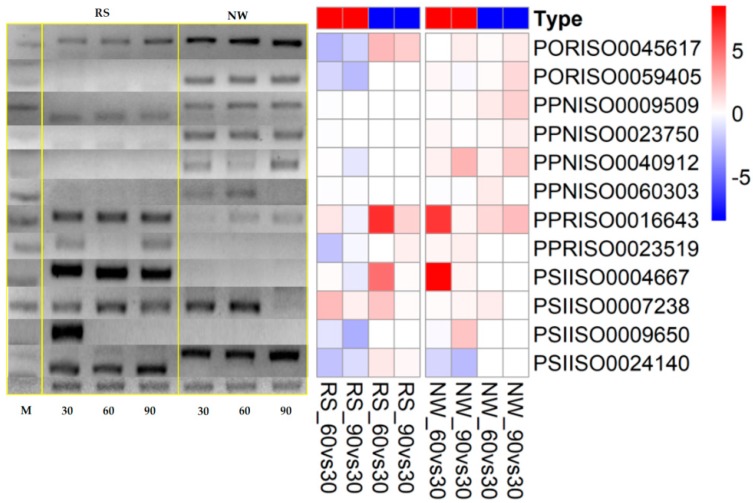
qRT-PCR validation of twelve transcripts. The M is the marker and the color code for the type is RNA-Seq (Red) and qRT-PCR (Blue) respectively. In the heat-map the relative expression of qRT-PCR and log_2_FC values were compared to assesses the similar expression. Here, the 16s rRNA is used as a house keeping in qRT-PCR experiment (bottom row of the gel-image).

In the heat map of the secondary metabolite biosynthesis transcripts, the BIA pathway (map0050) has shown a different coverage to other pathways in the *Papaver* transcriptome and to the other representative genomes (Figure 3). Naturally, *Papaver* species are known to be rich in alkaloid drugs [7,20,21,29], and, therefore, we have explored the BIA KEGG ortholog heat map (Figure 5). Furthermore, to understand the expressions of metabolite and the transcripts with the reference of KEGG pathway, the corresponding transcripts and metabolites were mapped into the BIA pathway using Pathview v.3.7 [36] (Figure 6) and their detailed metabolite quantification and relative differential expressions were plotted in the separate heat-maps by transforming the intensity values into *Z*-score (Figure 7). The comparative BIA heat map (Figure 5) includes 69 transcripts for 30 KEGG orthologs (KO). Among those, 49 transcripts were differentially expressed during the plant growth (Figure 2E). From the BIA co-occurrence heat map (Figure 5), we observed that four KOs ((*S*)-norcoclaurine synthase (K13382), (*S*)-scoulerine 9-*O*-methyltransferase (K13397), (*S*)-(styopine/canadine/nandinine) synthase (K13395), and reticuline *N*-methyltransferase (K21580)) are found in three genomes (*Macleaya cordata*, *Aquilegia coerulea*, and *Papaver* spp.), two KOs ((*S*)-cheilanthifoline synthase (K21070) and methyltetrahydrotoberberine 14-monooxygenase (K21692)) are found in *Papaveraceae* genomes (*Macleaya cordata* and *Papaver*), and three KOs (1,13-dihydroxy-*N*-methylcandine 13-*O*-acetyltransferase (K22095), salutaridine synthase (K13391), and *N*-methylcanadine 1-hydroxylase (K20621)) are only found in the *Papaver* transcriptome. Furthermore, some KOs are highly specific to single genomes, i.e., columbamine O-methyltransferase (K13398) is specific to *Aquilegia coerulea*, monoamine oxidase (K00274) is specific to *Medicago truncatula*, and aromatic-l-amino-acid (K01593) is specific to *Physcometrella patens*. Along with this heat-map, 22 alkaloids that are available with the known standards were quantified with LC-QTOF-MS/MS (Figure 7). In the metabolite quantitative analysis, we observed that *Papaver nudicaule* has a higher quantity of these alkaloids than *Papaver rhoeas* (Figure 7A), and an individual differential content of metabolites was noted (Figure 7B). In both species, the alkaloids from morphine to tetrahydropapaverine were highly accumulated in 90 days plant, and other metabolites have shown an opposite pattern in the *Papaver rhoeas* and *Papaver nudicaule* (Figure 7B). Furthermore, these collective profiles were marked with the BIA KEGG pathway diagram for an easy interpretation (Figure 6). The twelve transcripts that belong to BIA KOs were selected for the qRT-PCR to conform the similar expression patterns (Figure 8). From the validation, results show the similar expression from both the experiments, and although the values from the heterogenic experiment and statistical methods. These results might facilitate a hypothesis-driven experimental design for the detailed characterization of various secondary metabolite biosynthesis pathways and *Papaver* spp. plant associated genetic developmental problems. 

### 2.5. Cytochrome Transcripts 

**Figure 9:**
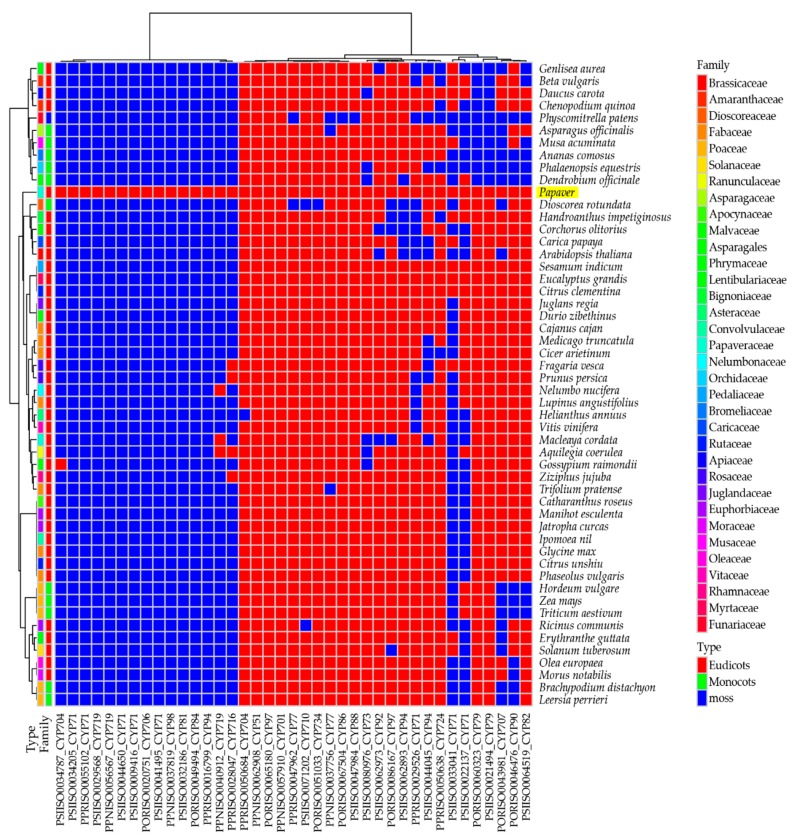
Full-length cytochrome existence profile. The expression of the heat map is shown in red (transcripts present) and blue (transcripts absent), this information obtained from the ortholog clusters.

**Figure 10:**
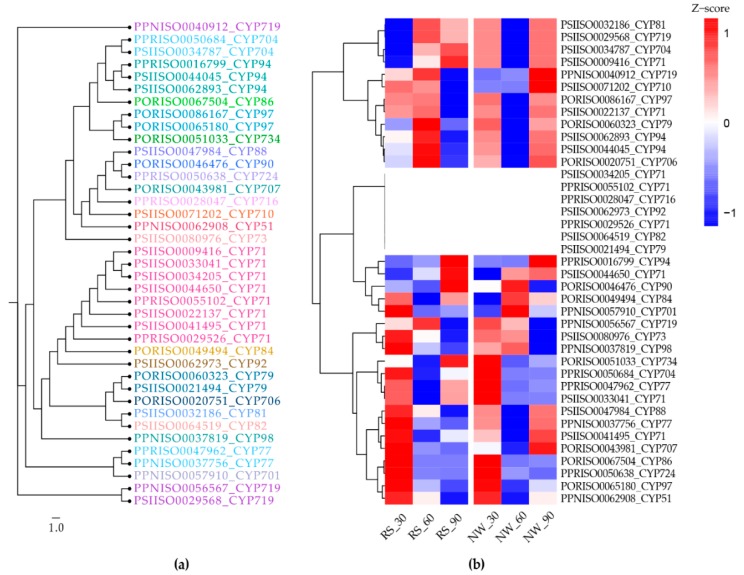
Cytochrome similarity and expression profile: (**a**) phylogenetic tree for the 39 full-length cytochromes; and (**b**) the corresponding expression profiles for 39 cytochromes. This tree was re-constructed from 1000 bootstrap trees.

The cytochrome heat map includes 39 transcripts of 23 cytochrome super families (Figure 9). Although 171 transcripts encoded for cytochrome domains, here, we have considered only the full-length transcripts. Further, to understand the cytochrome diversity, we have reconstructed a phylogenetic tree from 39 cytochromes (Figure 10A) and the corresponding expression profiles were given in the heat map (Figure 10B). Furthermore, seven cytochromes are not expressed in leaves, whereas they are expressed in seeds (thus, to prepare the representative transcriptome, we have included the seed transcriptome library). Moreover, we do not have the tissue-specific expression profile to claim that these cytochromes are specific to seeds. In our transcriptome, the cytochromes similar to families 71, 51, 86, 88, 716, 94, and 81, which play critical roles in species-specialized terpene biosynthesis in other plants, and the cytochrome families Cyp724, 734, 86, 90 and 94, which are involved in steroidal alkaloid and saponin biosynthesis in other plants, were also found in our transcriptome [37,38,39]. The Cyp719 cytochrome family was only present in alkaloid rich genomes, such as *Papaver* spp., *Aquilegia coerulea*, and *Macleaya cordata*. Furthermore, the two Cyp719 transcripts, along with seven other cytochromes, were only present in *Papaver* spp. Finally, the Cyp716 was only present in five genomes and it was shown to be crucial for tri-terpenoid biosynthesis [40]. These cytochrome profiles could aid in the production of more detailed functional characterizations (i.e., which cytochromes are specific to *Papaver* spp., also involved in BIA biosynthesis).

## 3. Discussion

Iso-quinoline alkaloids (BIA) are structurally diverse plant metabolites and include ~2500 different natural chemicals that have been partially characterized for their medicinal properties. Moreover, the BIA group contributes the most expensive drugs for the wellbeing of humanity, namely, codeine, morphine, sanguinarine, berberine, and noscapine [41]. Notably, these drugs are synthesized from the *Ranuncuales* order, which is becoming a model organism from which to engineer the BIA biosynthesis pathway [27,28,41]. However, very few genomes of *Ranuncuales*, are sequenced in public databases. In the *Papaveraceae* family, only *Macleaya cordata* has a draft genome sequence [33] with structural annotations, and other species have only transcriptome libraries. However, most of those libraries were prepared with Illumina, and none of those genomes has a PacBio sequenced transcriptome library and have previously been assessed for complete secondary metabolite profiling.

In our study, a 302 Mb (120,926 contigs) transcriptome was generated for *Papaver* ornamental species (*Papaver rhoeas*, and *Papaver nudicaule*), including 1185 (82.3%) complete BUSCO embryophyta core genes (Table 1). Although there are only few transcriptome libraries that are available for *Papaver* species, none of them were constructed with long read sequencing. Furthermore, the reliability of the functional annotations completely depends on their similarity scores only. In our transcriptome, 65% of the annotated transcripts hold similarity scores of more than 60–100%, which suggests that they are reliable and they could support more detailed functional characterizations (Figure 1B). Furthermore, the comparative analysis with 51 other genomes gives an overview of the secondary metabolite transcripts/genes concerning KEGG pathways (Figure 3). In *Papaver*, the phenylpropanoid biosynthesis pathway (map01061) and isoquinoline alkaloid biosynthesis pathway (map00950) shows different coverage when compared to other genomes (Figure 3), suggesting that different groups of alkaloids and phenylpropanoids could be present in *Papaveraceae* family genomes.

A few of the enzymes involved in the biosynthesis of noscapine, berberine, protopine, sanguinarine, and magnoflorine are only present in these three genomes (*Papaver*, *Macleaya coerulea*, and *Aquilegia coerulea*) and have different expression patterns concerning *Papaver* plant development (Figure 5). The transcripts present in these three genomes are involved in different types of alkaloid biosynthesis, for example, the (*S*)-norcoclaurine synthase (K13382) is involved in the initial step of codeine and morphine biosynthesis with specific “Pictet-Spengler” catalytic function, which is characterized from the genome *Thalictrum flavum* [42]. Additionally, this enzyme belongs to the pathogenesis-related (PR)-10/Bet v1 family [43]. (*S*)-scoreline 9-*O*-methyltransferase (K13397) and (*S*)-stylopine synthase are involved in the biosynthesis of sanguinarine, allocryptopine, and berberine. These enzymes have been characterized in *Eschscholzia californica* (California Poppy) and *Macleaya cordata* [44,45,46]. Reticuline *N*-methyltransferase (K21580) is involved in the magnoflorine biosynthesis and this has been characterized in *Papaver somniferum* [47]. Among these enzymes, three were regulated differently with respect to the growth and the species. Two enzymes ((*S*)-cheilanthifoline synthase (K21070) and methyl-tetra-hydro-protoberberine 14-monooxygenase (K21692)) were only shown to be present in the two genomes (*Macleaya cordata* and *Papaver*) that are involved in the biosynthesis of protopine and sanguinarine [48,49]. Finally, the two enzymes were shown to be specific to the *Papaver* genome, 1,13-dihydroxy-*N*-methylcandine 13-*O*-acetyltransferase (K22095), and *N*-methylcanadine 1-hydroxylase (K20621), which are involved in the biosynthesis of noscapine [50,51]. *N*-methylcanadine 1-hydroxylase is a rate-limiting factor for noscapine production. However, these transcripts are more highly expressed in young tissues than in mature tissues. Moreover, salutaridine synthase (K13391) is involved in the morphine biosynthesis, which is characterized by specific catalysis of the C–C phenol-couple in the isoquinoline class of alkaloids [52]. The validation of these transcripts shows similar pattern, although the quantification measures from different scale (Figure 8). Supporting the transcript’s expression characteristics, the alkaloids’ expression profiles were mapped in the BIA pathway for easy interpretation. Collectively, the transcriptome, along with the expression profiles and quantitative metabolite profiles, should provide basic observations of *Papaver* species (*Papaver rhoeas*, and *Papaver nudicaule*) secondary metabolites and their molecular function concerning plant growth stages. Moreover, these profiles could help to further detail the functional characterization of the various secondary metabolite biosynthesis and *Papaver* plant developmental associated genetic problems. The cytochrome families are found specific to the *Papaver* were Cyp 71, 719, 706, 98, 81, and 84, and these need detailed characterization to facilitate understanding of their relationships with the biosynthesis of alkaloids and other secondary metabolites (Figure 7). As in the cytochrome profile, there are few cytochromes, which are not expressed in the leaves, which results reflect the significance of organ-specific expression profiles. To obtain a complete gene profile and to understand the complete biosynthesis of alkaloids in *Papaver* spp., the organ-specific expression profiles indeed. Collectively, these expression profiles might facilitate a hypothesis-driven experimental design to provide a method for the sustainable production of *Papaver*-specific drugs. Furthermore, this could help biologists to elucidate the novel secondary metabolite biosynthesis.

## 4. Materials and Methods 

### 4.1. Plant Samples

The *Papaver* species *Papaver rhoeas*, and *Papaver nudicaule* were grown separately in the multiple pots at a controlled room temperature for three months. At three time points (30, 60, and 90 days), individuals were selected for sampling. The leaves of individual plants were collected for both the experiments, i.e., transcriptome sequencing and targeted metabolome analysis. The samples that were collected for the transcriptome were immediately frozen using the liquid nitrogen and then stored at −70 °C in a deep freezer. Additionally, the seeds from *Papaver nudicaule* were also collected for the PacBio sequencing. For each species, the experiments were repeated three times under the same conditions to obtain the biological replicates. 

### 4.2. PacBio Iso-Seq Library Preparation and Sequencing

All of the experimental procedures carried out in this project strictly followed the standard protocols provided in the respective product manuals. Total RNA was isolated from the frozen samples using a Qiagen RNA isolation kit (Qiagen Inc., Valencia, CA, USA). The mRNA from the total RNA was converted into cDNA with the Clontech SMARTer PCR cDNA synthesis kit (Takara Biomedicals, Tokyo, Japan) and amplified with large-scale PCR. Successfully-amplified PCR products were subjected to size selection (1–2 Kb, 2–3 Kb, 3–6 Kb) with the BluePippin system (Saga Science, Beverly, MA, USA), and these fractions were used for the library preparations. The SMRTbell library was constructed with the SMRTbell™ Template Prep Kit 1.0 (Pacific Biosciences, Menlo Park, CA, USA) and subsequent steps (i.e., DNA polymerase (DNA/polymerase binding kit P6); attached to the MagBeads (MagBead Kit)) were performed before the sequencing. All the prepared libraries were loaded into zero-mode waveguides (ZMWs) and sequenced with C4 chemistry with a 1 × 240 min movie was captured for each smart cell (DNA sequencing Reagent 4.0) using the PacBio RS (Pacific Biosciences) sequencing platform. Eight SMRT cells (1–2 Kb: 2 cells, 2–3 Kb: 2 cells, 3–6 Kb: 4 cells) were sequenced for each sample. The complete procedure was conducted by DNA Link, Seoul, South Korea.

### 4.3. PacBio Sequence Assembly and Annotation

All raw reads from each sample were subjected to the SMRT Analysis v2.2 RS_IsoSeq.1 (Pacific Biosciences) classification protocol to identify the high- and low-quality reads. Furthermore, the reads were clustered into isoforms and polished to obtain improved high-quality consensus reads using the TOFU pipeline (Pacific Biosciences) [53]. Since there is no reference genome for any *Papaver* species available, the obtained reads were re-clustered using CD-HIT-EST [54] with default parameters to remove the redundant isoforms. Finally, to obtain unique representative transcriptome sequences, all the sample sequenced reads were pooled together and clustered using CD-HIT-EST with the default parameters. These representative transcriptomes were annotated with BLAST2GO v.4 [55] using the Plant UniProt database (Updated 2018_02, https://www.uniprot.org/). Finally, the completeness of the reference transcriptome was assessed by the BUSCO method with the embryophyta (ODB9, created date: 13 February 2016) core gene dataset [32]. 

### 4.4. Illumina Library Preparation and Sequencing

Total RNA was isolated from the frozen samples with the Qiagen RNA isolation kit, and mRNA in the total RNA was converted into library templates using TruSeq RNA Sample Prep Kit v2 (Illumina, San Diego, CA, USA). This procedure includes purification, synthesis of the strands, end repair, adapter ligation, and PCR enrichment. Enriched libraries were quantified and used for sequencing with Illumina Hi-Seq 4000 (Illumina). Finally, the sequenced reads were processed with the sequencing control software, and the outputs were paired-end with the fastq formatted files. The complete procedure was conducted by Macrogen Inc, Seoul, Korea.

### 4.5. LC-QTOF-MS/MS Metabolome Analysis 

Two-gram homogenized sample taken from the total leaves sample were mixed with 5 mL of ethanol and subjected to the sequential steps—vortexing and sonication—to obtain the concentrated (20 mg/mL) extract. Prepared samples were subjected to liquid chromatography-mass spectrometry with a Thermo Scientific Vanquish UHPLC system (ThermoFisher Scientific, CA, USA) with Poreshell 120 PFP (2.1 × 100 mm, 2.7 μm), column (Agilent), and a triple ToF 5600^+^ mass spectrometer system (Triple ToF MS) (SCIEX, Foster City, CA, USA) with a Duospray^TM^ ion source and universal gradient conditions. The system was calibrated before the original experiment with the blank samples and 18 alkaloid standards. Molecular weights and molecular breakdown information detected from the system were collected through the information-dependent acquisition (IDA) scan method in LC-QTOF (HRMS, high-resolution mass spectrometer). Finally, all the peaks for the given standards and similar predictable alkaloids component peaks were obtained and were annotated with Metlin (https://metlin.scripps.edu/) and isotope MS. All of the experimental procedures were conducted at the Korean Medicine Clinical Trial Center. The multivariate analysis of the data was conducted using R (https://www.r-project.org/).

### 4.6. Differential Gene Expression

Complete short read sequences were preprocessed using Trimmomatic v0.36 [56] to remove low-quality chimeric sequences and adapter contaminants. Processed reads were mapped to the reference transcriptome (prepared from the PacBio assembly) using Bowtie2 v.2.3.3 [57]. Concordantly mapped reads were quantified using RSEM [58] to obtain the reads per kilo-base per million (RPKM) and TPM scores for each library. The transcripts per kilo-base million (TPM) values were subjected to edgeR [59] to obtain the differential expression patterns for the individual groups.

### 4.7. KEGG Secondary Metabolite Biosynthesis Proteins

To obtain the existence profile of the KEGG secondary metabolite biosynthesis transcripts of the *Papaver* transcriptome, the KEGG reference dataset was prepared from the KEGG pathway database (https://www.genome.jp/kegg/pathway.html) from two major categories, the metabolism of terpenoids and polyketides and the biosynthesis of other secondary metabolites. The complete protein sequences were obtained for the KEGG maps by following relationships (maps → KEGG orthologs → Swiss/Uniprot). These protein sequences were the reference dataset for the KEGG database, mainly for the characterized genes for secondary metabolite biosynthesis. With respect to the KEGG secondary metabolite biosynthesis existence profile, Figure 4 shows the color gradients, which represent the normalized values of each KEGG pathway. The value that was calculated as the normalized value = (number of KEGG orthologs (KO) has similar transcripts to the reference transcriptome/total number of KO in a given pathway) × 100. This normalized value was plotted in a heat map (KEGG maps vs. genomes). In the other existence profiles, the color codes are the binary representations (present: red and absent: blue).

### 4.8. Ortholog Analysis and Phylogeny Construction

In this study, ortholog analyses were conducted to place the *Papaver* transcriptome in different clusters that were based on the available genomes from the different functional groups. These classified groups are as follows: edible fruits, different colored flowers, crops/oil seeds, and Ensembl plant genomes. The credibility of the functional annotations and the existence of the genes with a sequence similarity context were assessed. Out of the 52 genomes included in the ortholog analysis, 17 genomes were from Ensembl plants (https://plants.ensembl.org), and *Catharanthus roseus* downloaded from medicinal plant genomic resources (http://medicinalplantgenomics.msu.edu), and the others were from Genbank assemblies (Supplementary Table S1). The complete protein sequences from genomes were trimmed based on the sequence length (between 10 and 3000 AA). Furthermore, the selected sequences were subjected to ortholog analysis using the OrthoMCL [60] method with default parameters. The single copy genes were aligned using the MAFFT v7.2 [61] with default parameters. The multiple alignments were initially corrected with Gblocks v0.91 [62] and were used for phylogenetic tree construction by IQ-TREE v1.5.0 [63]. The tree was imported to FigTree v1.4.3. (http://tree.bio.ed.ac.uk/software/figtree/) to obtain an image (Figure 2). 

### 4.9. Cytochrome Family Analysis

The complete transcripts were compared with the CYPED (https://cyped.biocatnet.de/) [64] database using the CD-HIT method (to obtain CYP family), with the parameters S:60 and C:60. The full-length cytochrome transcripts were obtained with the Transdecoder method (https://github.com/TransDecoder/TransDecoder). The full-length cytochromes were compared with other selected genomes from ortholog clusters. The cytochromes were aligned using MAFFT v7.2 [61] with default parameters. Multiple alignments were initially corrected with Gblocks v0.91 [62] and were used for phylogenetic tree construction by IQ-TREE v1.5.0 [63]. The tree was imported to FigTree v1.4.3. (http://tree.bio.ed.ac.uk/software/figtree/) to obtain an image (Figure 7).

### 4.10. qRT-PCR for Ramdom Selected Transcripts

The mRNA were isolated from the leaves samples, similar to RNA sequence library preparation in method Section 4.4. The mRNAs are converted to cDNA using M-MuLV reverese transcriptase (Merck KGaA, Darmstadt, Germany). Those PCR products were prepared for SYBR Green based protocol and further subjected to CFX96^TM^ Bio-Rad qRT-PCR instrument (Bio-Rad, Hercules, CA, USA) and the downstream data analysis for cycle threshold (CT) and relative expressions were performed with CFX manager (Ver. 3.1, Bio-Rad). The transcripts specific primers are designed through primer3 method (http://bioinfo.ut.ee/primer3-0.4.0/). The list of primers given in Table 2.

### 4.11. Data Submission

The complete mRNA sequences were submitted to the GenBank-SRA repository under the project identification number PRJNA476004.

## Figures and Tables

**Table 1:** Quantitative summary of transcriptome along with annotations (**A**) sequencing and assembly; (**B**) functional annotation; (**C**) translation summary of the transcriptome; and, (**D**) completeness assessment of the transcriptome.

A. Sequencing and Assembly				
Technology	PacBio (Iso-Seq)		Illumina (Hi-Seq)	
	Reads	Bases (MB)	Reads	Bases (GB)
Raw Sequence (HQ * Reads)	324,602	700	1,273,902,514	128.8
Processed Sequence	324,602	700	1,235,970,946	125.8
Denovo Assembled Contigs (N50)	120,926 (3117)	302		
Reference Mapped			79.7% (Average of mapped reads)	
Expressed	59,135			
**B. Annotations**				
	**# of Sequences**		**# of Reference IDs**	
BLAST Hits	93,672 (77.5%)		29,862	
Gene Ontology	69,399 (57.4%)		3587	
KEGG Enzymes	13,187 (11%)		675	
**C. Transdecoder**				
Total Transcripts	103,043 (85.2%)			
Complete	83,384 (68.9%)			
5′ Partial	15,913 (13.1%)			
3′ Partial	3664 (3%)			
Internal	82			
**D. BUSCO**				
Total Core Genes	1440 (100%)			
Complete	1185 (82.3%)			
Fragmented Core Genes	75 (5.2%)			
Missing Core Genes	180 (5.2%)			

* HQ: PacBio high-quality consensus reads.

**Table 2:** List of primers used for qRT-PCR.

Transcripts	Forward	Reverse
PORISO0045617	GACAATGAACAAGGATACC	CTTCTTCTGCTTCGTCTA
PORISO0059405	CTTCCATTGTTGCTCTTCTACTTG	TGCTGCTGCTGTTGATGA
PPNISO0009509	ATCGTGTGAAGGAGATAC	TTAGGACCAGTGCTTATG
PPNISO0023750	AACCACCACCAAGATACC	GGCGATAAGCAACTAATGTC
PPNISO0040912	GCTGTTAGAGATGTGGAA	CTTCTTCGTCGTAATTCTG
PPNISO0060303	TTATGGTGGCAAGATGAGT	TGTTGTTCGGTCCAGTAA
PPRISO0016643	TCCATTATCAGCCAGTTC	TCTCCGCTTATGTAATCG
PPRISO0023519	GATTCTCGCATTCGGATT	ATCACCATTGGATCTTGTC
PSIISO0004667	CTTGTGTTCTTGATGGGAAA	CGATAAGGCTAGGCAGAT
PSIISO0007238	TGGATACTTGGCATCTTG	GTGGCTTACATCTTCCTT
PSIISO0009650	CCAAGGAACTCTTCATCA	CTTGCGTTCATTAGACTTAC
PSIISO0024140	CAATGGAGAAGAATGGATGA	GTAACACAAGGAAGGATGAA

## Supplementary Materials

Supplementary materials can be found at http://www.mdpi.com/1422-0067/19/10/3192/s1.
